# 1,6-Dehydropinidine Is an Abundant Compound in *Picea abies* (Pinaceae) Sprouts and 1,6-Dehydropinidine Fraction Shows Antibacterial Activity against *Streptococcus equi* Subsp. *equi*

**DOI:** 10.3390/molecules25194558

**Published:** 2020-10-06

**Authors:** Virpi Virjamo, Pia Fyhrquist, Akseli Koskinen, Anu Lavola, Katri Nissinen, Riitta Julkunen-Tiitto

**Affiliations:** 1 Natural Product Research Laboratory, Department of Environmental and Biological Sciences, University of Eastern Finland, P.O. Box 111 Joensuu, Finland; juhoakseli@hotmail.com (A.K.); anu.lavola@uef.fi (A.L.); katri.nissinen@uef.fi (K.N.); riitta.julkunen-tiitto@uef.fi (R.J.-T.); 2 School of Forest Sciences, University of Eastern Finland, P.O. Box 111 Joensuu, Finland; 3 Division of Pharmaceutical Biosciences, Faculty of Pharmacy, University of Helsinki, P.O. Box 56 Helsinki, Finland; pia.fyhrquist@helsinki.fi

**Keywords:** pinaceae, norway spruce, *Picea abies*, *Streptococcus equi*, equine strangles, antibacterial, piperidine, alkaloid

## Abstract

Knowledge about the defensive chemistry of coniferous trees has increased in recent years regarding a number of alkaloid compounds; in addition to phenolics and terpenes. Here, we show that Norway spruce (*Picea abies* (L.) H. Karst.), an important boreal zone tree species; accumulates 1,6-dehydropinidine (2-methyl-6-(2-propenyl)-1,6-piperideine) in its needles and bark. We reanalyzed previously published GC-MS data to obtain a full picture of 1,6-dehydropinidine in *P. abies*. 1,6-dehydropinidine appeared to especially accumulate in developing spring shoots. We used solid-phase partitioning to collect the alkaloid fraction of the sprouts and thin-layer chromatography to purify 1,6-dehydropinidine. The antibacterial properties of the 1,6-dehydropinidine fraction were tested using a broth microdilution method; with *Streptococcus equi* subsp. *equi* as a model organism. Based on our results 1,6-dehydropinidine is common in alkaloid extractions from *P. abies* (0.4 ± 0.03 mg g^−1^ dw in mature needles) and it is especially abundant in young spruce shoots (2.7 ± 0.5 mg g^−1^ dw). Moreover; 1,6-dehydropinidine extracted from *P. abies* sprouts showed mild antibacterial potential against *Streptococcus equi* subsp. *equi* (MIC 55 µg mL^−1^). The antibacterial activity of a plant compound thought of as an intermediate rather than an end-product of biosynthesis calls for more detailed studies regarding the biological function of these coniferous alkaloids

## 1. Introduction

*Pinus sylvestris* L. and *Picea abies* (L.) H. Karst. are dominant tree species in northern boreal forests. Recently, there here has been increasing interest in the secondary chemistry of *P. sylvestris* and *P. abies* as a source of bioactive compounds [1]. Specifically, the wood extractives of these species have shown to have antibacterial properties against methicillin-resistant *Staphylococcus aureus* and *Escherichia coli* [2] and *P. abies* resin-based salve has been proven to be clinically effective for the treatment of chronic wounds [3]. The defensive chemistry of coniferous species consists of all three major secondary chemistry groups—phenolics (e.g., acetophenones, lignans, stilbenes, flavonoids, phenolic acids and proanthocyanidins), terpenes (e.g., monoterpenes and resin acids) and alkaloids (piperidines) but the bioactive compounds responsible for antimicrobial properties have been assumed to be phenolic and terpene compounds [1]. Piperidine alkaloids are traditionally considered more important in plant-herbivore interactions than in microbial defense [4,5]. Recently, the role of alkaloids in microbial interactions has, however, attracted increasing attention (e.g., Reference [6]) which raises the question of the biological role of alkaloids in conifers as well.

**Figure 1:**
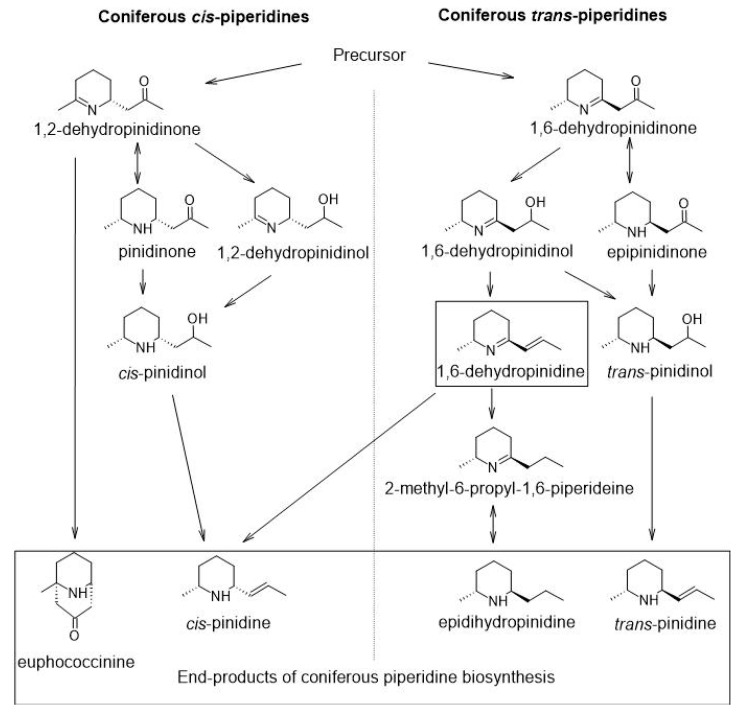
Proposed biosynthesis of coniferous 1,6-desubtituted piperidines according to References [4,10,12,13,14].

The first 2,6-disubstituted piperine found in conifers was *cis*-pinidine, named after the *Pinus* genus [7]. Since then, a number of other compounds with slight modifications on the basic 9-carbon body with an N-containing heterocyclic ring have been found, including double bonds, hydroxyl groups, oxygen groups in the ring or side chain and *cis*/*trans* isomers [4,8,9]. Only four of the compounds are considered end-products of biosynthesis: *cis*-pinidine and euphococcinine from *cis*-pathway and *trans*-pinidine and epidihydropinidine from the *trans*-pathway [10] (Figure 1). Different coniferous genera have different main components. *Pinus* species typically accumulate either *cis*-pinidine or euphococcinine, while in *Picea* species, both *cis* and *trans* end-products occur simultaneously [4]. In *P. abies,* epidihydropinidine is the most abundant alkaloid compound in adult needles, while *cis*-pinidinol is a major compound in bark [8,11].

Piperidine alkaloids usually show mass-spectra with a mass peak from 139 to 169 [4]. However, some coniferous alkaloid fractions have also shown an unidentified compound with a probable molecular weight of 137 [15,16], indicating a double bond both in the ring structure and in the side chain. Recently, Veličkovic et al. [13] identified this compound with mass spectrometry imaging as 1,6-dehydropinidine. We have suggested that based on its structure, this compound [16] would be an intermediate of the *trans*-pathway leading to the biosynthesis of epidihydropinidine [10]. However, Veličkovic et al. [13] proposed that in the roots of *Pinus strobus* L. and *Pinus taeda* L., 1,6-dehydropinidine would be a direct precursor of *cis*-pinidine (Figure 1). Because 1,6-dehydropinidine has not been identified until recently, there are no quantitative data on its abundance.

In this study, we investigated alkaloid fractions of *P. abies* for the presence of 1,6-dehydropinidine. Moreover, because environmental and developmental factors and genetic background are known to affect the defensive secondary chemistry of trees [17,18], we re-investigated our previously published coniferous alkaloid data [11,12,19,20] to obtain an overview of the abundance of 1,6-dehydropinidine in *P. abies*. As identification of the 1,6-dehydropinidine [13] was reported after publication of these data sets, the compound has not been included in the original publications. 1,6-dehydropinidine was further purified from crude alkaloid extract for antibacterial activity analysis. Here, *Streptococcus equi* subsp. *equi* was selected as a model organism for investigating the role of 1,6-dehydropinidine as an antibacterial agent. *S. equi* is a gram-positive bacteria that causes equine strangles disease, resulting in severe financial losses in the horse industry [21]. Recently, notable proportion of isolated *S. equi* subsp. *equi* have shown resistance to first-choice antibiotics [22]. Based on this data, the role of 1,6-dehydropinidine in piperidine alkaloid biosynthesis and *P. abies* defense is discussed.

## 2. Results

### 2.1. Abundance of 1,6-Dehydropinidine in P. abies

1,6-dehydropinidine (2-methyl-6-(2-propenyl)-1,6-piperideine) was found to be present nearly in all studied *P. abies* alkaloid datasets investigated. When concentrations were calculated as epidihydropinidine equivalents, 1,6-dehydropinidine was one of the most abundant piperidine alkaloid compounds in *P. abies*, accounting for, on average, 42.3% (±1.4 SEM) of total piperidine alkaloid yield. In mature needles, the concentration of 1,6-dehydropinidine was, on average, 0.4 mg g^−1^ (±0.03 SEM, dry weight [dw]).

**Figure 2:**
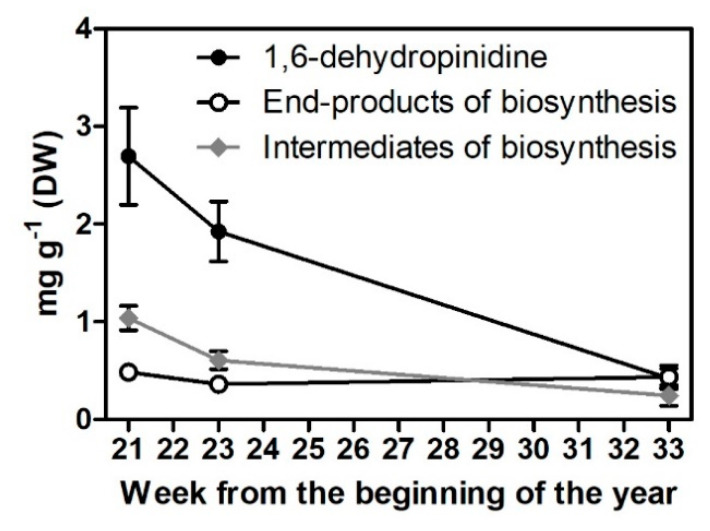
Change in 1,6-dehydropinidine concentration from developing needles (weeks 21–23) to mature needles (week 33). Data for intermediates and end-products of piperidine alkaloid biosynthesis are from Reference [12]. Here, *cis*-pinidinol is considered an end-product of biosynthesis because detectible amounts of *cis*-pinidine were not recorded.

We detected significant variations in the concentration of 1,6-dehydropinidine according to some variables (Table 1). There was no difference between 1,6-dehydropinidine concentrations in bark and needle samples but the genetic background of the tree (tree origin), as seen in the data from a provenance experiment, had a significant effect (Table 1), although there was no clear relationship between geographical origin and 1,6-dehydropinidine concentration. The highest concentration of 1,6-dehydropinidine was detected from *P. abies* trees with genetic backgrounds in Latvia and the lowest concentration was detected in trees with backgrounds in Northern Finland (Table 2). A clear effect on 1,6-dehydropinidine concentrations was detected for needle developmental stage (needle age, Table 1). In young developing needles, the highest concentrations of all studied GC-MS (gas-chromatography coupled with mass spectrometry) datasets, 2.7 mg g^−1^ (±0.5 SEM, dw) of 1,6-dehydropinidine, were recorded. In immature needles, 66% of total alkaloids consisted of 1,6-dehydropinidine (Figure 2). In terms of environmental conditions, +33% enhanced ultraviolet-B radiation, +2 °C increased temperature and current year fertilization [19] did not show any effect on 1,6-dehydropinidine abundance in mature needles. The appearance of 1,6-dehydropinidine seems to be clearly linked to other alkaloid compounds assumed to be early intermediates of biosynthesis (Table 3, Figure 1). Notably, when reanalyzing the previously published GC-MS dataset [11,12,19,20] in which 1,6-dehydropinidine was not included in total alkaloid concentrations, no changes to the original conclusions were made and no detectable amounts of 1,6-dehydropinidine were found in the previously published *P. sylvestris* data [23].

### 2.2. Antibacterial Activity of 1,6-Dehydropinidine Fraction

**Figure 3:**
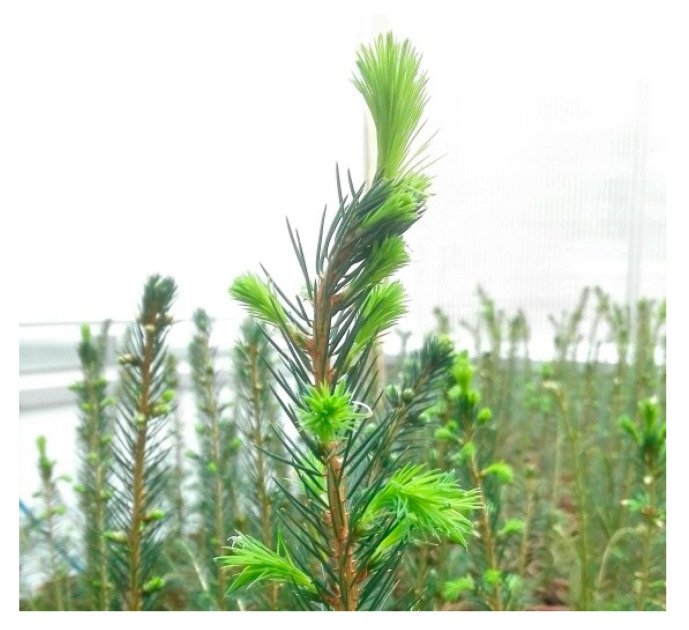
Young spruce sprouts used as a starting material for 1,6-dehydropinidine extraction. (Photograph Virpi Virjamo).

**Figure 4:**
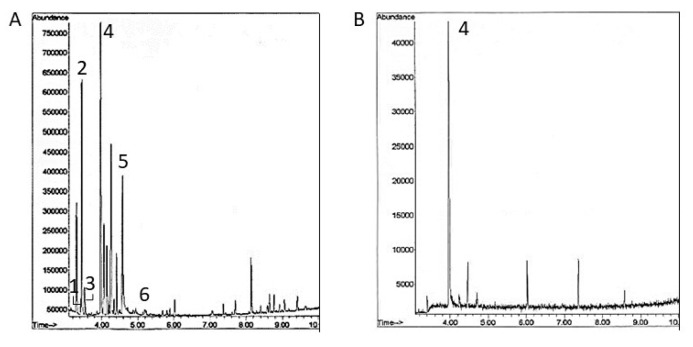
Examples of GC-MS runs (first 10 min of the 20 min run shown) for (**A**) the crude alkaloid fraction and (**B**) purified 1,6-dehydropinidine fraction. Numbers 1-6 refers to 2-methyl-6-propyl-1,6-piperideine, epidihydropinidine, *trans*-pinidine, 1,6-dehydropinidine, epipinidinone + *cis*-pinidinol, and, tentative, 1,6-dehydropinidinone, respectively. Other peaks are monoterpene and simple alkane impurities and were not quantified.

Because the highest 1,6-dehydropinidine concentrations were found in young spruce sprouts (Figure 2), these were used as a starting material for the purification of the 1,6-dehydropinidine fraction (Figure 3). After thin-layer chromatography, the 1,6-dehydropinidine fraction, purified from the crude alkaloid fraction, showed no detectable amounts of other piperidine alkaloid compounds in the GC-MS run (Figure 4B). However, the method used in the purification was not complete, because only 0.22 mg of 1,6-dehydropinidine was obtained from 28 g of fresh spruce sprouts.

**Figure 5:**
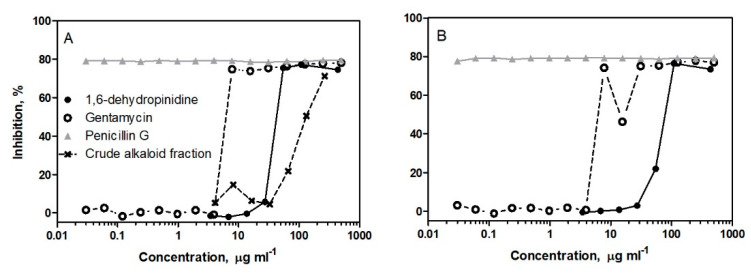
The inhibition activity (%) of the 1,6-dehydropinidine fraction and control antibiotics (gentamycin and penicillin G) against *S. equi* after (**A**) 24 h and (**B**) 48 h incubation in Mueller-Hinton broth. The inhibition of the crude alkaloid fraction of sprout alkaloids after 24 h is presented as a reference for 1,6-dehydropinidine. The concentrations of the studied compounds are expressed on a log10 scale.

**Figure 6:**
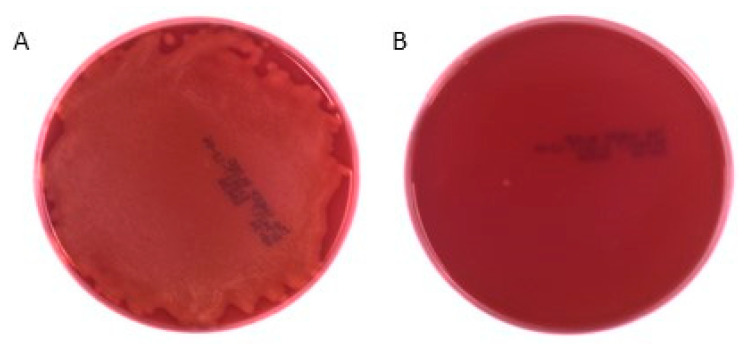
Growth of *S. equi* after 24h incubation with (**A**) 55 µg mL^−1^ and **(B**) 110 µg mL^−1^ of 1,6-dehydropinidine fraction on blood agar. Bacterial growth in (**A**) is visible as lighter colored area covering almost whole plate. Plates are photographed without lid and black markings are from commercial plates. (Photograph Akseli Koskinen).

In this study, the antibacterial activity of 1,6-dehydropinidine was investigated against the equine strangles caused by the bacterium *S. equi* subsp. *equi* using both Mueller-Hinton broth without blood supplement and blood agar plates. The efficiency of 1,6-dehydropinidine was compared to the inhibition efficiency of the crude total alkaloid fraction (including 2-methyl-6-propyl-1,6-piperideine, epidihydropinidine, *trans*-pinidine, 1,6-dehydropinidine, epipinidinone, *cis*-pinidinol and tentative 1,6-dehydropinidinone, Figure 4A) and commercial antibiotics, penicillin G and gentamycin. At a concentration of 55 µg ml ^−1^ and using the broth microdilution method, 1,6-dehydropinidine inhibited the growth of *S. equi* by over 70% after 24 h as compared to a growth control but only by 22% after 48 h (Figure 5). The total alkaloid fraction inhibited the growth of *S. equi* by 70–80% with a concentration of 265 µg mL^−1^. Gentamycin inhibited 75% of the growth of *S. equi* with a concentration of 7.8 µg mL^−1^, whereas penicillin still inhibited 78% of growth, even with the lowest tested concentration of 0.03 µg mL^−1^. On the blood agar petri dishes, the 1,6-dehydropinidine fraction completely inhibited the growth of *S. equi* at a concentration of 110 µg mL^−1^, which could be considered the minimum bactericidal concentration (MBC) of this alkaloid (Figure 6).

## 3. Discussion

Based on our results, 1,6-dehydropinidine is an important part of the alkaloid group in *P. abies*. Its appearance is tightly linked with the abundance of other piperidine alkaloids and it is especially abundant in young *P. abies* sprouts. Our results regarding the timely correlation with other early *cis* and *trans* piperidines (Table 3) support the assumption that 1,6-dehydropinidine plays a role in the early biosynthesis of piperidine alkaloids [10]. *P. abies* does not accumulate *cis*-pinidine in the needles or bark or it does so in concentrations too low to detect [11,19]. Thus, based on this data, the other proposed role of 1,6-dehydropinidine, as an immediate precursor of *cis*-pinidine [13], cannot be discussed.

1,6-dehydropinidine concentrations did not change in response to environmental factors such as increased temperature, fertilization or enhanced ultraviolet-B radiation. This further supports its role in the *trans*-piperidine pathway, because the abundance of *cis*-piperidines in P. *abies* has tended to increase at elevated temperature [19] and that in *Pinus ponderosa* has been shown in increase with fertilization (Douglas ex C. Lawson) [17]. Similar to another intermediate compound in the *trans*-pathway, 2-methyl-6-propyl-piperideine [11], 1,6-dehydropinidine seems to occur in remarkable concentrations in mature needles. This could be a result of the constant biosynthesis of *trans*-pathway piperidines in *P. abies* needles or the specific role of these compounds for *P. abies*, for example, in defense against herbivores or pathogens.

The extraction efficiency for the purified 1,6-dehydropinidine was low in this experiment. This is partly due to the fact that some thin-layer chromatography fractions were rejected due to their containing impurities or low concentrations of the focus compound. However, it is possible that an unnecessarily large part of these volatile alkaloids may have been lost in repeated solid-phase partitioning (SPP), although generally, SPP is considered a suitable extraction method for coniferous alkaloids [11,24]. In further studies, a more efficient extraction system should be applied.

In this study, the growth of the equine strangles-causing bacterium *S. equi* was, in some case, inhibited by the 1,6-dehydropinidine fraction purified from *P. abies* sprouts. To the best of our knowledge, this is the first time that 1,6-dehydropinidine has been tested for its antibacterial activity. Growth inhibition took place, however, at notably higher concentrations for 1,6-dehydropinidine (MIC 55 µg mL^−1^) as compared to the tested commercial antibiotics (penicillin G MIC < 0.03 µg mL^−1^ and gentamycin MIC 7.8 µg mL^−1^). It is worth noting that more experiments investigating 1,6-dehydropinidine and its growth-inhibitory effects against *S. equi* should be performed with blood-enriched media, as is recommended for use with streptococci [25]. The use of these media might result in higher MIC values because *S. equi* would grow more optimally with blood enrichment. On the other hand, the use of optical density to measure bacterial growth would be difficult if a blood-enriched medium were used instead of the relatively clear Mueller-Hinton broth used in our study.

The inhibition of the growth of *S. equi*, however, did take place at smaller concentrations for 1,6-dehydropinidine than for the crude alkaloid fraction (Figure 5). At 133 µg mL^−1^, the crude alkaloid fraction caused a 50% inhibition of the growth of *S. equi* (where 48 µg mL^−1^ of detected alkaloids were 1,6-dehydropinidine) and at 265 µg mL^−1^, the crude alkaloid fraction caused 71% inhibition (where 95 µg mL^−1^ of detected alkaloids were 1,6-dehydropinidine). Thus, the antibacterial activity of the crude alkaloid fraction could be partly explained by the inhibition activity of 1,6-dehydropinidine, although the other alkaloids present in the fraction (2-methyl-6-propyl-1,6-piperideine, epidihydropinidine, *trans*-pinidine, epipinidinone, *cis*-pinidinol and tentative 1,6-dehydropinidinone) should be tested separately.

There are only a few prior publications reporting antibacterial activity for coniferous alkaloids. Euphococcinine has shown weak activity against gram-negative bacteria but *cis*-pinidinol was found to lack antibacterial activity in early attempts to clarify the role of coniferous alkaloids [4]. Synthetic (±)epidihydropinidine has shown a very weak antibacterial effect (MIC 5.37 to 43 mg mL^−1^ against selected gram-positive, gram-negative and fungal micro-organism) [26]. Based on our results, 1,6-dehydropinidine may be the most promising candidate among *P. abies* piperidine compounds for further, antibacterial screenings using a wider range of strains, while the role of other related compounds, such as *cis*-pinidine, considered highly toxic for vertebrates, might solely act in defense against herbivores [4,8]. This could suggest that in addition to the end-products (epidihydropinidine, *trans*-pinidine, euphococcinine and *cis*-pinidine), intermediates in alkaloid biosynthesis may play an important biological role for conifers.

The young sprouts of *P. abies*, in which the highest 1,6-dehydropinidine concentration was detected, are considered to be pro-health in traditional medicine and have been used for the treatment of cough among other symptoms [27]. The relatively mild antibiotic activity detected here against the gram-positive bacterium *S. equi* does not support the notion that 1,6-dehydropinidine would be the main actor among the bioactive compounds in spruce sprouts. Thus, the interaction between 1,6-dehydropinidine and other bioactive compounds in sprouts should be considered in further studies. Young *P. abies* sprouts have a high concentration of proanthocyanidins [12] and they accumulate many kaempherol derivatives, whereas picein and many lignans are rare in sprouts, though they are abundant in mature needles [28]. Similarly, the monoterpene concentration of *P. abies* needles reaches its maximum late in the growth season [29]. The synergism of alkaloids and phenolics generating stronger antibacterial activity together than separately has been reported for *Berberis* extractions [30]. This raises the question whether synergistic or additive interactions on the part of these compounds (phenolics, terpenes and alkaloids) could be important for the chemical defense of *P. abies* against micro-organisms, including plant pathogenic bacteria, especially in vulnerable, still developing *P. abies* shoot structures.

## 4. Materials and Methods 

### 4.1. Identification of 1,6-Dehydropinidine (2-methyl-6-(2-propenyl)-1,6-piperideine)

The alkaloid fraction was extracted from the *P. abies* needles with a solid-phase partition (SPP) according to Reference [19]. Shortly, approximately 1 g of homogenized plant material was incubated in 17 mL 0.5 M aqueous HCl for 1 h, filtered and then made basic (pH ≥ 11) with 6 M aq. NaOH. Alkaloids were then separated from other compounds with Extrelut^®^ NT 20 PE columns (Merck KGaA, Darmstadt, Germany) using SupraSolv^®^ dichloromethane (Merck) as an eluent. Eluents were concentrated with a Laborota 4002-digital rotary evaporator (Heidolph, Schwabach, Germany) and sample composition was analyzed with a gas-chromatography mass spectrometer (GC-MS, Agilent 6890, Santa Clara, CA, USA, see details on conditions used in Reference [11]) and a direct infusion mass spectrometry (bypass of ultrahigh pressure liquid chromatography) with quadrupole time-of-flight (QTOF)-MS (1200, Agilent Technologies, see details for used conditions from Reference [31]). For QTOF-MS analyses, dichloromethane was dried with a rotary evaporator and the alkaloid fraction was re-diluted to 1 mL of 99.5% ethanol (ETAX Aa, Altia, Helsinki, Finland). The identification of the unknown compound in the alkaloid fraction with a GC-MS retention time of 3.97 min and a mass peak of 137 was based on comparison with the literature values [13,15,16]. 1,6-dehydropinidine: GC-MS ions 70 eV *m*/*z* (relative intensity, %), [M]^+^ 137 (36), 122 (100), 94 (23), 84 (26) 68 (61). QTOF-MS *m*/*z* 138.1278 (M + H), mass measurement error −0.729 ppm.

### 4.2. Abundance of 1,6-Dehydropinidine in P. abies

We reanalyzed GC-MS runs from several previously published datasets for piperidine alkaloids, revealing the occurrence of 1,6-dehydropinidine in *P. abies* and the conditions affecting it. Specifically, GC-MS data from Reference [11] were used to reveal differences in the abundance of 1,6-dehydropinidine between needles and bark. Also, Reference [19] was used to analyze the effects of temperature, fertilization and UV radiation on the occurrence of 1,6-dehydropinidine. Data from a provenance experiment [20] was used to reveal genetic variation in the accumulation of 1,6-dehydropinidine in *P. abies*. Finally, the original data from Reference [12] was used to follow the accumulation of the compound during the development of new sprouts. All 1,6-dehydropinidine concentrations were calculated as (±)epidihydropinidine equivalents [11]. Statistical analyses were conducted with IBM SPSS statistics Version 25 (Armonk, New York, NY, USA). A univariate ANOVA with Tukey’s HSD as a post-hoc test was used for a comparison of multiple groups (needle age or tree origin as a fixed factor). Data was log10 transformed to meet the assumptions of a parametric test. For the comparison of two parallel groups (plant part, UV-radiation, temperature and fertilization as fixed factor), a Mann-Whitney U Test was employed because the assumptions of parametric tests were not met even after the transformations. Accordingly, Spearman’s rho was used to test the correlation between the abundance of 1,6-dehydropinidine and other identified coniferous piperidine alkaloids.

### 4.3. Isolation of 1,6-Dehydropinidine from P. abies Sprouts

Alkaloids were extracted from seed-originated, commercial 2-year-old nursery-grown *P. abies* seedlings (61°N 26°E, Fin Forelia Oy, Finland). Seedlings were grown until the new shoots emerged in a greenhouse of the University of Eastern Finland, Department of Environmental and Biological Sciences, (62°N 29°E, Joensuu, Finland) in spring 2017. Sprouts were then harvested in plastic bags and stored at −18 °C until further processing during winter 2018. The extraction of the crude alkaloid fraction was performed with SPP as above, by modifying the amount of solvent used given the larger amounts of preparative material. A total of 28 g of fresh sprouts were used for the extraction. 1,6-dehydropinidine was purified from the crude alkaloid fraction with thin-layer chromatography (TLC). Silica plates (PLC Siliga gel 60 F254, 2mm, Merck KGaA, Darmstad, Germany) were used, with 6:3:1:0.1 ethyl acetate:methanol:MQ-H_2_O:25% ammonium as the liquid phase. After the TLC, a section of plate was colored with iodine and the corresponding sections were separated from the plates to 20 mL of 0.5 M aq. HCl. After 15 min of incubation, the pH was changed to basic (pH ≥ 11) with 6 M aq. NaOH and alkaloids were extracted from the sample with SPP, as with the crude alkaloid fraction. The alkaloid fractions were then concentrated with a rotary evaporator and analyzed with GC-MS, the fractions containing 1,6-dehydropinidine were pooled and concentrations were calculated as (±)epidihydropinidine equivalents [11].

### 4.4. Antibacterial Activity of 1,6-Dehydropinidine Against Streptococcus Equi Subsp. Equi

For antibacterial analyses, both the crude alkaloid fraction from the sprouts (containing 13% 2-methyl-6-propyl-1,6-piperideine, 17% epidihydropinidine, 9% *trans*-pinidine, 36% 1,6-dehydropinidine, 13% epipinidinone+*cis*-pinidinol and 11% tentative 1,6-dehydropinidinone, identification with GC-MS according to Reference [12] (Table S1 in Supplementary Materials) and concentrations calculated as (±)epidihydropinidine equivalents [11]) and purified 1,6-dehydropinidine fraction were studied. Dichloromethane fractions obtained directly from SPP (crude alkaloid fraction) or from combined TLC extractions (1,6-dehydropinidine fraction) were dried with a rotary evaporator and re-eluted to 150 µL (crude alkaloid fraction) or 250 µL (1,6-dehydropinidine fraction) MQ-H_2_O and stored in +8 °C until the analyses. Antibacterial analyses intended to obtain MIC (minimum inhibitory concentration) values were conducted against *Streptococcus equi* subsp. *equi* ATCC 9528 using a microdilution method [32]. Gentamycin (Sigma-Aldrich/Merck KGaA, Darmstadt, Germany) and penicillin G (Medix BioChemica, Espoo, Finland) were used as controls for antimicrobial activity. Bacterial suspensions were grown overnight at +37 °C, 100 RPM in Mueller-Hinton broth and diluted to 1 × 10^6^ CFU mL^−1^. 100 µL of this diluted bacterial suspension were added to the 96-well microplate wells and 100 µL of 1,6-dehydropinidine (3.4–439 µg mL^−1^), crude alkaloid extract (4.1–265 µg mL^−1^) or control antibiotics (0.03–500 µg mL^−1^) diluted with Mueller-Hinton broth, were added to the wells. Thus, the final number of bacterial cells at T0 of the test was 5 × 105 CFU mL^−1^ in the microplate wells. Turbidity was measured at 620 nm using a spectrophotometer (Victor, Wallac, Finland) after 24 h and 48 h incubations at +37 °C, 100 RPM (for crude alkaloid extract only 24h value was obtained). The MIC values were estimated after a growth period of 24 h as % growth inhibition values compared to the growth control. A 70–80% growth inhibition was considered as the MIC because these growth inhibition percentages resulted in no visible growth. Moreover, the maximum growth inhibition % for penicillin and gentamicin was 80%, even for high concentrations, presumably due to the slow growth of *S. equi* in Mueller-Hinton medium without blood enrichment. The percentage growth and growth inhibition were calculated according to the following formulas:

(1) $$\%\;\mathrm{growth}\;=\frac{\mathrm{mean}\;\%\;\mathrm{growth}\;\mathrm{of}\;\mathrm{test}\;\mathrm{well}\;\mathrm{duplicates}}{\mathrm{mean}\;\%\;\mathrm{growth}\;\mathrm{of}\;\mathrm{growth}\;\mathrm{control}\;\mathrm{wells}\;}\times \;100.$$

% inhibition of growth = 100 − % growth.(2)

Minimum bactericidal concentration values for 1,6-dehydropinidine, gentamycin and penicillin G were obtained either by continuing the incubation of the microplate so that total incubation time was 48 h or by transferring 100 µL of the bacterial suspension from the MIC-wells of the microplate to a petri dish (BBL™ Trypticase™ Soy Agar with 5% Sheep Blood (TSA II), Becton, Dickinson and Company, Sparks, MD, USA) that was incubated overnight at +37 °C. A clear petri dish after incubation indicated that the used concentration of 1,6-dehydropinidine or antibiotics killed the bacterium and thus also indicated the MBC values of these compounds.

## 5. Conclusions

Here, we show that 1,6-dehydropinidine is an important compound in *P. abies*’ alkaloid chemistry, especially in young developing needles. Based on our results, researches should consider re-visiting previously studied *Picea* and *Pinus* species for the appearance of 1,6-dehydropinidine. Moreover, the antibacterial activity of 1,6-dehydropinidine against gram-positive *S. equi* challenges the notion that the end-products of biosynthesis are the most important bioactive compounds. In further studies of the biological role of coniferous alkaloids, the intermediates of biosynthesis should also be considered as potentially active compounds.

## Figures and Tables

**Table 1:** Summary of the effect of plant part, needle age, tree origin and environmental factors on 1,6-dehydropinidine concentrations in *P. abies*.

Factor	*p*-Value	Effect	Data Reference
Plant part (bark vs. needles)	0.653 ^1^		[11]
**Needle age (first visible, developing, mature)**	**0.000** ^2^	**↑ in early needles**	[12]
**Tree origin (12 origins, see Table 2)**	**0.009** ^2^	**no clear pattern**	[20]
UV-radiation (ambient vs. +33% UVB)	0.093 ^1^		[19]
Temperature (ambient vs. +2 °C)	0.937 ^1^		[19]
Fertilization (ambient vs. fertilized)	0.310 ^1^		[19]

^1^ Mann-Whitney U Test, ^2^ ANOVA (log10 transformed); statistically significant results highlighted in bold.

**Table 2:** 1,6-dehydropinidine concentration in mature needles of *P. abies* from provenance experiment in which trees of different origins were grown for 35 years in the same place (N°61, E°29).

Origin		mg g^−1^ (dw)	Tukey′s HSD
Northern Finland	**N68°E24°**	**0.16 ± 0.03**	**a**
	N67°E23°	0.25 ± 0.04	ab
Central Finland	N63°E29°	0.33 ± 0.04	ab
	N64°E28°	0.34 ± 0.04	ab
Southern Finland	N61°E23°	0.26 ± 0.04	ab
	N60°E23°	0.24 ± 0.03	ab
Russia	N61°E40°	0.25 ± 0.06	ab
Latvia	**N56°E25°**	**0.38 ± 0.05**	**b**
Poland	N53°E23°	0.29 ± 0.04	ab
Slovakia	N48°E20°	0.20 ± 0.04	ab
France	N48°E6°	0.29 ± 0.05	ab
Romania	N47°E25°	0.19 ± 0.03	ab

Concentration is reported as average ±SEM. GC-MS (gas-chromatography coupled with mass spectrometry) data are reanalyzed from Reference [20]. Statistically significant results of Tukey’s HSD (honestly significant difference) highlighted in bold.

**Table 3:** Results of correlation analyses (Sperman’s rho) for 1,6-dehydropinidine concentration with other alkaloid compounds detected from current-year needles.

Piperidine Alkaloid Compounds from *P. abies*		Correlation Coefficient
Early *trans*-piperidines	**1,6-dehydropinidinone**	**0.605** **
	**epipinidinone**	**0.480** **
	**1,6-dehydropinidinol**	**0.671** **
Early *cis*-piperidines	**pinidinone**	**0.758** **
	**1,2-dehydropinidinone**	**0.663** **
	1,2-dehydropinidinol	0.082
Late *trans*-piperidines	2-methyl-6-propyl-1,6-piperideine	0.190
	epidihydropinidine	0.094
	*trans*-pinidine	0.054
	*trans*-pinidinol	0.072
Late *cis*-piperidines	**euphococcinine**	**0.769** **
	*cis*-pinidinol	−0.10

“Early” and “late” refer to the assumed position in the alkaloid biosynthesis pathway (Figure 1). Statistically significant results are highlighted in bold (** for *p* < 0.01). Data for compounds other than 1,6-dehydropinidine are from Reference [12].

## Supplementary Materials

The following are available online, Table S1: Retention order in GC and MS-fragmentation pattern of alkaloids in crude alkaloid fraction.
