# Supplementary material for: 1,6-Dehydropinidine Is an Abundant Compound in Picea abies (Pinaceae) Sprouts and 1,6-Dehydropinidine Fraction Shows Antibacterial Activity against Streptococcus equi Subsp. equi
Source: Molecules. 2020 Oct 6;25(19):4558. doi: 10.3390/molecules25194558 (PMC7582581; doi:10.3390/molecules25194558)
Supplement: Supplementary file 1 [file molecules-25-04558-s001.pdf]

Table S1. Retention order in GC and MS-fragmentation pattern of alkaloids in crude alkaloid fraction. Main fragment for each compound is marked bold.

Compound	Retention time (min)	EI mass spectrum, m/z (%)
2-methyl-6-propyl-1,6-piperideine	3.40	139 (21) [M+], 124 (58), 111 (53), **96 (100)**, 83 (17), 70 (43), 55 (16), 44 (21), 42 (20), 41 (21)
epidihydropinidine	3.44	141 (1) [M+], 126 (7), 99 (6), **98 (100)**, 81 (5), 70 (6), 56 (7), 55 (8), 43 (5), 41 (5)
trans-pinidine	3.51	139 (20) [M+], **124 (100)**, 96 (51), 84 (33), 82 (59), 68 (54), 49 (33), 42 (28)
epipinidinone+*cis*-pinidinol	4.53+4.57	142 (10), 138 (10), 134 (10), **98 (100)**, 96 (12), 94 (11), 93 (19), 82 (20), 81 (13), 71 (13), 70 (17), 69 (26), 56 (16), 55 (28), 44 (21), 43 (25), 41 (13), 40 (9)
tentative 1,6-dehydropinidinone	5.32	153 (11) [M+], 110 (15), **96 (100)**, 94 (16), 70 (16), 42 (10), 41 (9)
